# Antiparasitic nitazoxanide potentiates colistin against colistin-resistant *Acinetobacter baumannii* and *Escherichia coli in vitro* and *in vivo*

**DOI:** 10.1128/spectrum.02295-23

**Published:** 2023-11-30

**Authors:** Mengxin Xu, Zhuocheng Yao, Jingchun Kong, Miran Tang, Qi Liu, Xiaotuan Zhang, Shiyi Shi, Xiangkuo Zheng, Jianming Cao, Tieli Zhou, Zhongyong Wang

**Affiliations:** 1 Department of Clinical Laboratory, The First Affiliated Hospital of Wenzhou Medical University, Key Laboratory of Clinical Laboratory Diagnosis and Translational Research of Zhejiang Province, Wenzhou, China; 2 Department of Clinical Laboratory, Laboratory Medicine Center, Zhejiang Provincial People’s Hospital (Affiliated People’s Hospital), Hangzhou Medical College, Hangzhou, Zhejiang, China; 3 Department of Medical Lab Science, School of Laboratory Medicine and Life Science, Wenzhou Medical University, Wenzhou, China; Emory University School of Medicine, Atlanta, Georgia, USA

**Keywords:** colistin-resistant, synergistic effect, drug repurposing, nitazoxanide, *Acinetobacter baumannii*, *Escherichia coli*

## Abstract

Multidrug-resistant Gram-negative bacterial infections pose a serious threat to public health and safety. Colistin is a line of defense against these infections, but colistin-resistant (COL-R) bacteria have emerged worldwide. This study aimed to explore the synergistic potential of nitazoxanide (NTZ) in combination with colistin for the treatment of COL-R *Acinetobacter baumannii* and *Escherichia coli* strains *in vitro* and *in vivo*. The checkerboard and time-killing assays indicated that NTZ in combination with colistin exhibited potent antibacterial activity against COL-R *A. baumannii* and COL-R *E. coli*. The antibiofilm activity measured by crystal violet staining and scanning electron microscopy combined with a confocal laser scanning microscope revealed that the combination of NTZ and colistin could inhibit biofilm formation and eradicate the formed biofilm. This study also demonstrated that the application of this drug combination to an *in vivo* infection model significantly increased the survival rate of *Galleria mellonella* larvae. Hemolysis tests revealed that the combination of NTZ and colistin showed no cytotoxicity. Altogether, the combination of NTZ and colistin showed promising synergistic antibacterial and antibiofilm effects *in vitro* and *in vivo*. This combination might be used as an alternative therapy for treating COL-R *A. baumannii* and COL-R *E. coli* infections.

**IMPORTANCE:**

Colistin is used as a last resort in many infections caused by multidrug-resistant Gram-negative bacteria; however, colistin-resistant (COL-R) is on the rise. Hence, it is critical to develop new antimicrobial strategies to overcome COL-R. We found that nitazoxanide (NTZ) combined with colistin showed notable synergetic antibacterial activity. These findings suggest that the NTZ/colistin combination may provide an effective alternative route to combat COL-R *A. baumannii* and COL-R *Escherichia coli* infections.

## INTRODUCTION

The continuous emergence of multidrug-resistant (MDR) strains has become a global problem due to incorrect antimicrobial drug usage (1). Consequently, many antibiotics are no longer effective against even the simplest infections (2). Colistin (polymyxin E) has been recently reintroduced into clinical use, mainly to treat infections caused by MDR and extensive drug-resistant Gram-negative pathogens. However, with the overuse of colistin (COL), Gram-negative bacteria, including COL-R *A. baumannii* and COL-R *E. coli*, have gradually developed resistance to this “last-resort” antibiotic. This could become an uncontrollable global challenge.

Biofilm is an assemblage of microorganisms where the microorganisms adhere to a substrate and are encapsulated within a self-produced matrix comprising extracellular polymeric substances, proteins, and extracellular DNA (3). It can enhance bacterial tolerance to antibiotics and innate immunity, ensuring survival and reproduction (3). Biofilm inhibitors, either alone or in combination with commercial antibiotics, reduce bacterial adhesion, thus reducing infection persistence, eliminating biofilm infection, and reducing the risk of drug resistance development (4).

Nitazoxanide (NTZ) is an FDA-approved oral antiprotozoal drug with a good bioavailability and safety profile (5). It has been clinically applied for the treatment of giardia and cryptosporidium-associated diarrhea (6). NTZ inhibits pyruvate: ferredoxin/flavodoxin oxidoreductases, nitroreductases, and peptide disulfide isomerases. In addition, NTZ inhibits pyruvate oxidoreductase in *Helicobacter pylori*, anaerobes, parasites, and *Campylobacter jejuni* (7). Studies have shown that NTZ has potent antibacterial activity against Gram-positive bacteria such as *Staphylococcus aureus* and *Enterococcus* spp., including antibiotic-resistant strains, methicillin-resistant *S. aureus*, and vancomycin-resistant *S. aureus* (8).

To our knowledge, this study is the first to report the synergistic antibacterial and antibiofilm effects of NTZ combined with COL against COL-R *A. baumannii* and COL-R *E. coli*. This combination may offer a new therapeutic option for infections caused by COL-R pathogens.

## RESULTS

### Antimicrobial susceptibility testing

The minimum inhibitory concentrations (MICs) of different isolates against commonly used clinical antibiotics and NTZ are listed in Table 1. The MIC of COL against all strains was 4–32 µg/mL. These strains have well-defined COL-R mechanisms, which were identified by the experimental team (9, 10). The resistance mechanism of *E. coli* to COL was attributed to the *mcr-1* mobile COL resistance gene. COL resistance in *A. baumannii* was mediated by the substitution of LpxA, LpxC, LpxD, and PmrB and upregulation of the AdeABC/AdeIJK efflux pump. The MIC of all strains for NTZ was ≥256 µg/mL, which suggested that NTZ has no or low antibacterial activity against COL-R *A. baumannii* and COL-R *E. coli*. All bacterial isolates (16/16; 100%) were COL-R and displayed an MDR phenotype.

**TABLE 1:** MICs of commonly used clinical antibiotics and nitazoxanide against COL–R *A. baumannii* and COL–R *E. coli*

Species	Strains^*a*^	Antibiotics^*c*^									NTZ
		ATM	CAZ	FEP	IPM	CIP	LVX	GEN	TOB	COL	
		Breakpoints(S-R)^*b*^ MIC (μg/mL)									
		8–32	8–32	8–32	2–8	0.5–2	1–4	4–16	4–16	2–4	
	**ATCC25922**	0.06	0.12	0.016	0.06	0.008	0.008	0.5	1	1	
	**ATCC27853**	4	1	2	1	0.25	0.5	1	1	1	
*A. baumannii*	**BM2349**	16	**64**	**64**	**64**	**32**	**8**	**64**	**64**	**4**	≥256
	**BM2431**	**64**	**64**	**64**	**16**	**4**	**8**	1	1	**32**	≥256
	**BM2412**	16	**64**	**64**	**16**	**4**	**8**	4	1	**16**	≥256
	**BM2370**	8	**32**	**128**	**8**	**128**	**8**	4	1	**8**	≥256
	**BM2622**	**64**	**64**	**64**	**8**	**4**	**8**	**16**	**16**	**8**	≥256
	**BM1539**	16	8	**64**	**16**	**4**	2	1	1	**4**	≥256
	**BM1579**	16	16	4	2	**2**	**4**	**32**	**16**	**16**	≥256
	**BM2390**	**64**	**64**	**64**	**16**	**4**	**8**	**16**	1	**4**	≥256
		4–16	4–16	2–16	1–4	0.25–1	0.5–2	4–16	4–16	2–4	
*E. coli*	**DC3539**	**64**	**128**	**≥256**	0.5	**64**	**16**	**128**	**128**	**8**	≥256
	**DC3737**	**≥256**	**≥256**	**≥256**	**128**	**≥256**	**≥256**	**≥256**	**≥256**	**8**	≥256
	**DC3846**	**128**	**64**	**≥256**	0.5	**≥256**	**128**	**≥256**	**64**	**8**	≥256
	**DC4887**	1	4	**32**	1	**4**	**16**	**16**	8	**16**	≥256
	**DC5286**	**≥256**	**128**	**≥256**	0.25	**128**	**64**	4	4	**16**	≥256
	**DC7333**	**≥256**	**≥256**	**≥256**	**16**	**≥256**	**128**	**128**	**≥256**	**8**	≥256
	**DC8277**	4	≤1	≤1	≤1	**≥4**	**≥8**	**≥16**	**≥16**	**8**	≥256
	**DC8313**	4	**16**	≤1	≤1	**≥4**	**≥8**	≤1	≤1	**4**	≥256

^*a*^ Bolded strain number indicates multidrug resistant (MDR) strain.

^*b*^ S-R represents the susceptible (S) breakpoint to resistant (R) breakpoint, according to CLSI supplement M100 (30th edition) and EUCAST.

^*c*^ ATM, Aztreonam; CAZ, Ceftazidime; FEP, Cefepime; IMP, Imipenem; CIP, Ciprofloxacin; LVX, Levofloxacin; GEN, Gentamicin; TOB, Tobramycin; COL, colistin; NTZ, nitazoxanide.

### Synergistic antibacterial activity of COL and NTZ revealed by checkerboard assays

We investigated the antibacterial activity of NTZ against COL and NTZ. The MIC of COL and NTZ was 4–32 µg/mL and ≥256 µg/mL, respectively (Table 2). Checkerboard analysis showed that the combination of COL and NTZ exerted a synergistic effect on COL-R *A. baumannii* and COL-R *E. coli* strains with different COL-R mechanisms, with the fractional inhibition concentration index (FICI) ranging from 0.25 to 0.5 (Table 2). The presence of NTZ reversed the COL-R phenotype to the COL-S phenotype in all strains.

**TABLE 2:** FICI value for colistin/nitazoxanide combinations against COL–R *A. baumannii* and COL–R *E. coli* ^*a*^

Species	Strains	Monotherapy (μg/mL)		Combination (μg/mL)		FICI	Interpretation
		Colistin	Nitazoxanide	Colistin	Nitazoxanide		
*A. baumannii*	BM1539	4	≥256	0.5	64	0.375	Synergistic
	BM1579	16	≥256	0.5	64	0.281	Synergistic
	BM2622	8	≥256	1	64	0.375	Synergistic
	BM2349	4	≥256	1	64	0.5	Synergistic
	BM2370	8	≥256	0.5	64	0.312	Synergistic
	BM2412	16	≥256	0.5	64	0.281	Synergistic
	BM2431	32	≥256	0.5	64	0.266	Synergistic
	BM2390	4	≥256	0.5	64	0.375	Synergistic
*E. coli*	DC3539	8	≥256	2	32	0.375	Synergistic
	DC3737	8	≥256	2	32	0.375	Synergistic
	DC3846	8	≥256	2	32	0.375	Synergistic
	DC4887	16	≥256	2	32	0.25	Synergistic
	DC5286	16	≥256	2	32	0.25	Synergistic
	DC7333	8	≥256	2	32	0.375	Synergistic
	DC8277	8	≥256	2	32	0.375	Synergistic
	DC8313	4	≥256	1	32	0.375	Synergistic

^*a*^ FICI, fractional inhibitory concentration index.

### Impact on the formation and eradication of biofilms

**FIG 1:**
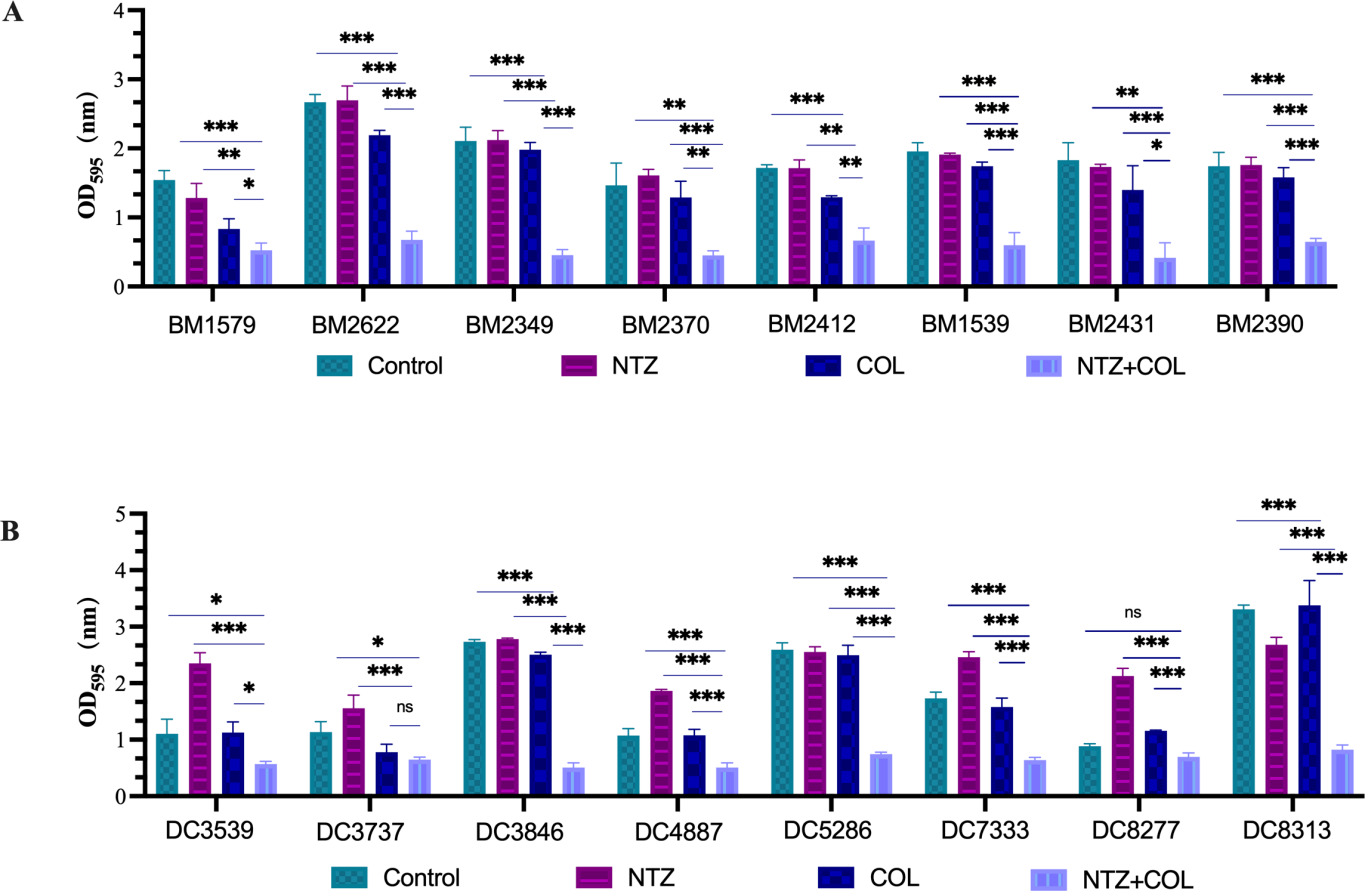
(**A**) Biofilm inhibitory effects of COL combined with NTZ on COL-R *A. baumannii*. (**B**) Biofilm inhibitory effects of COL combined with NTZ on COL-R *E. coli*. Data were analyzed by Student’s *t*-test (ns, not statistically significant, *P < 0.05, **P < 0.01, ***P < 0.001). OD_595_, optical density at 595 nm. All data are reported as the mean ± SEM (*n* = 3 group) from two independent experiments.

All the tested strains could form a biofilm. Hence, crystal violet staining was used to investigate the ability of NTZ and COL, alone or in combination, to inhibit biofilm formation. The combination of NTZ and COL significantly inhibited biofilm formation by COL-R *A. baumannii* and COL-R *E. coli* (Fig. 1). The synergistic concentration for each strain was determined using the checkerboard method. In COL-R *A. baumannii*, the selected NTZ concentration was 64 µg/mL, whereas COL concentrations were 0.25 µg/mL and 0.5 µg/mL (Fig. 1A). In COL-R *E. coli*, the selected NTZ concentration was 32 µg/mL and 64 µg/mL, whereas the COL concentration was 1 µg/mL (Fig. 1B).

**FIG 2:**
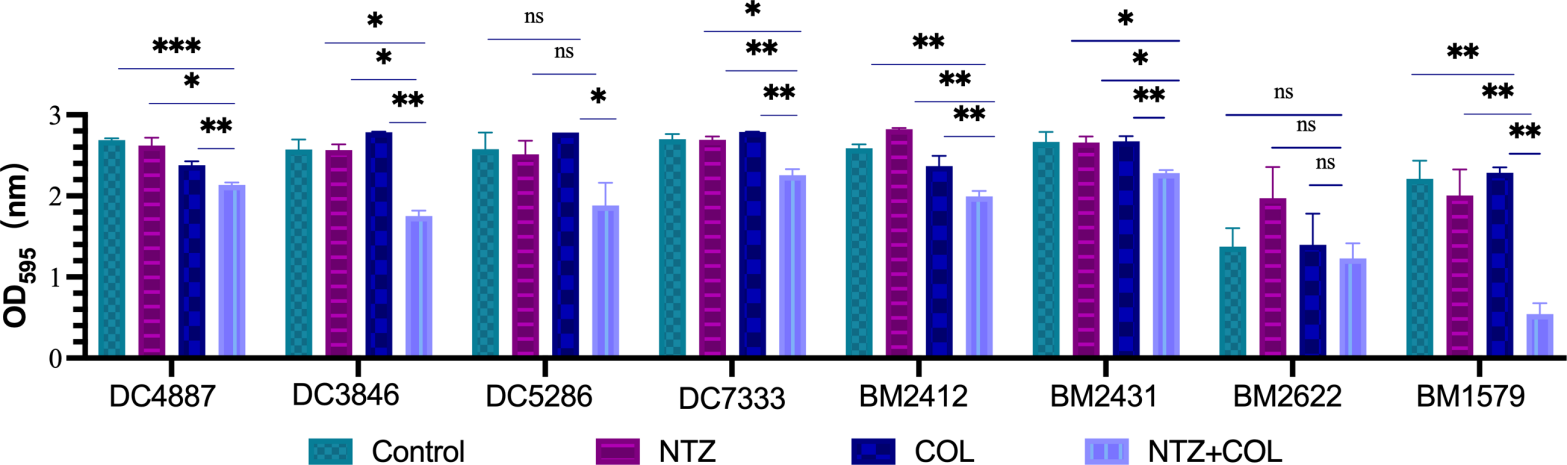
Eradication of COL-R *A. baumannii* and COL-R *E. coli* biofilms by COL and/or NTZ. Data were analyzed by Student’s *t*-test (ns, not statistically significant, *P < 0.05, **P < 0.01, ***P < 0.001). OD_595_, optical density was measured at 595 nm. All data are expressed as the mean ± SEM (*n* = 3 group) from two independent experiments.

The biofilm eradication assay results showed that the combination of 4 µg/mL COL and 128 µg/mL NTZ easily eradicated most tested strains (7/8) compared to the control and monotherapy groups (Fig. 2). In conclusion, the combination of COL and NTZ inhibited biofilm formation in COL-R *A. baumannii* and COL-R *E. coli* and also eradicated the established biofilms.

### The synergistic effect of COL and NTZ revealed by the time-kill assay

**FIG 3:**
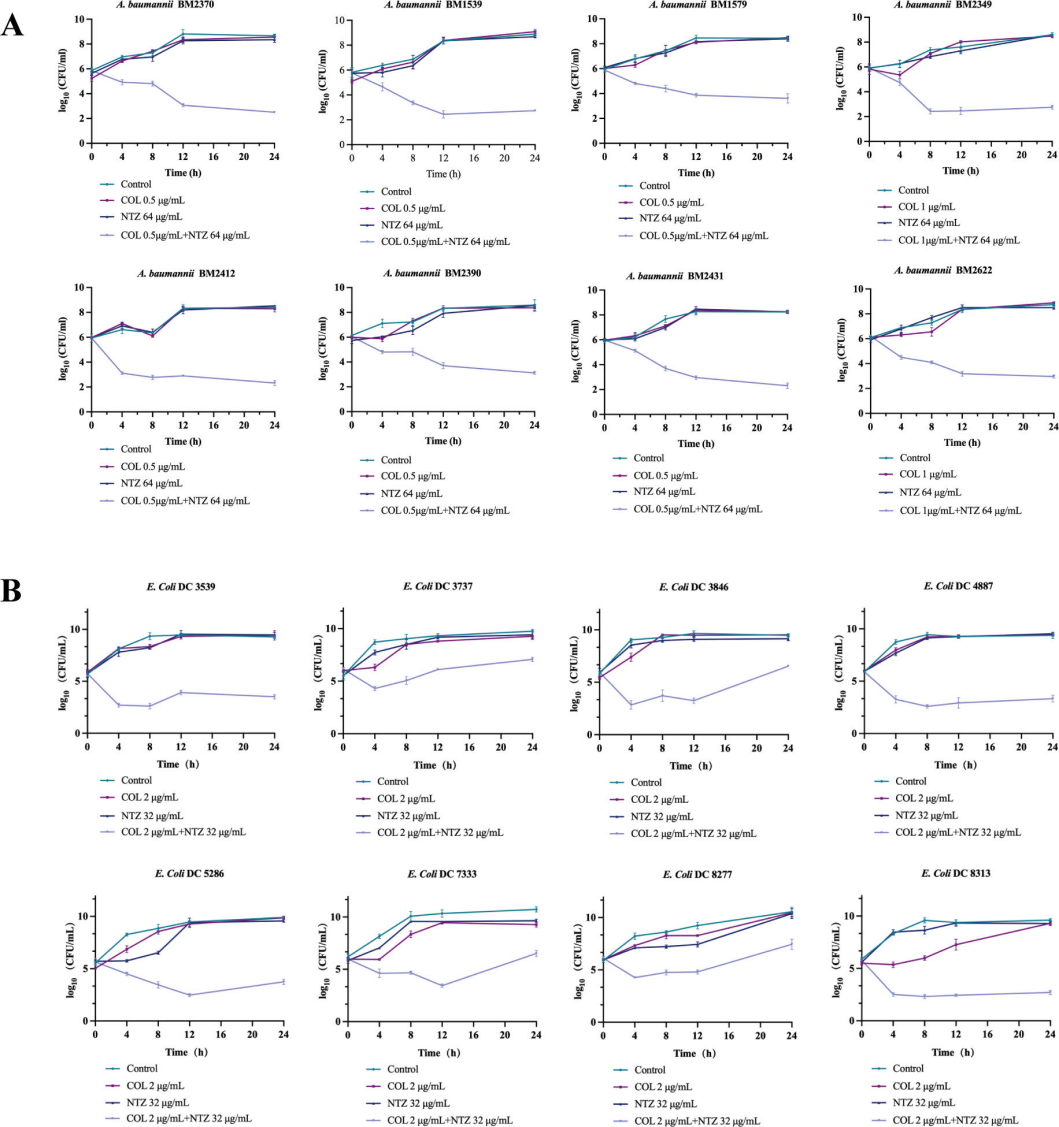
(A) Time-kill curves of COL and NTZ alone or in combination against COL-R *A. baumannii*. (**B**) Time-kill curves of COL and NTZ alone or in combination against Col-R *E. coli*. All data are reported as the mean ± SEM (*n* = 3 group) from two independent experiments.

Time-kill assays further confirmed the dynamic synergistic antibacterial effects of NTZ and COL on COL-R bacteria. The concentrations of these two drugs were determined using a checkerboard assay with FICI ≤0.5. In COL-R *A. baumannii*, the selected NTZ concentration was 64 µg/mL, whereas COL concentrations were 0.5 µg/mL and 1 µg/mL (Fig. 3A). In COL-R *E. coli*, the selected NTZ concentration was 32 µg/mL, whereas the COL concentration was 2 µg/mL (Fig. 3B). The growth of the tested strains could not be inhibited by either COL or NTZ within 12 h. However, the combination of NTZ and COL significantly decreased the bacteria load by approximately >3 log_10_ in colony-forming units (CFU)/mL compared to the other treated group within 24 h. Thus, regardless of the strain, bacterial suspensions treated with COL plus NTZ exhibited 2–6 log_10_ CFU/mL lower counts than the control group at the 24-h mark.

### Scanning electron microscopy (SEM) and confocal laser scanning microscope (CLSM) images

**FIG 4:**
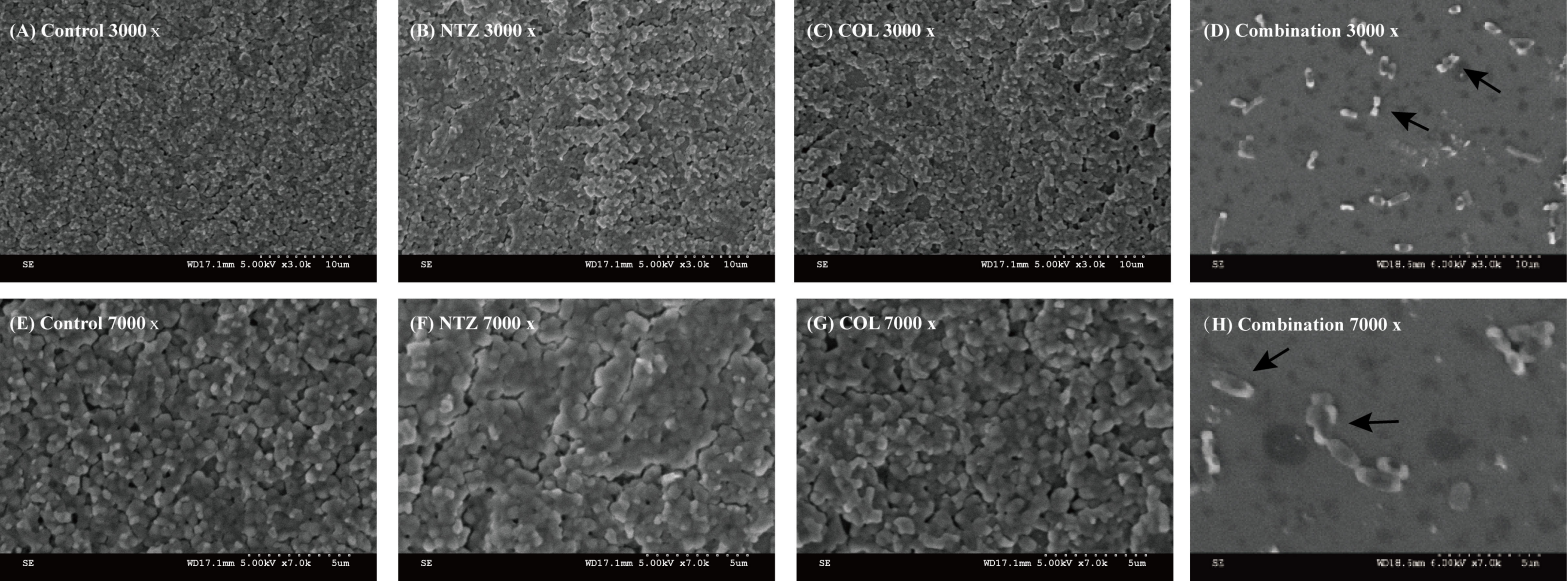
SEM images displaying the effect of different treatments on the number and structure of biofilm of COL-R *E. coli* DC3846. (**A**) LB broth control at ×3,000 magnification. (**B**) NTZ alone at ×3,000 magnification. (**C**) COL alone at ×3,000 magnification. (**D**) COL/NTZ combination at ×3,000 magnification. (**E**) LB broth control at ×7,000 magnification. (**F**) NTZ alone at ×7,000 magnification. (**G**) COL alone at ×7,000 magnification. (**H**) COL/NTZ combination at ×7,000 magnification.

To further explore the effect of the NTZ/COL combination on biofilm, we employed SEM to investigate the combination’s effects. The results are shown in Fig. 4. When observed at 3,000 and 7,000× magnification, cells in the control, NTZ monotherapy (32 µg/mL), and COL monotherapy (1 µg/mL) groups showed complete morphology and high density. The arrows in the combination group (Fig. 4D and H) point to cells that display bacterial cell morphology, cell shrinkage, and the appearance of vesicles, bulges, and wrinkles.

**FIG 5:**
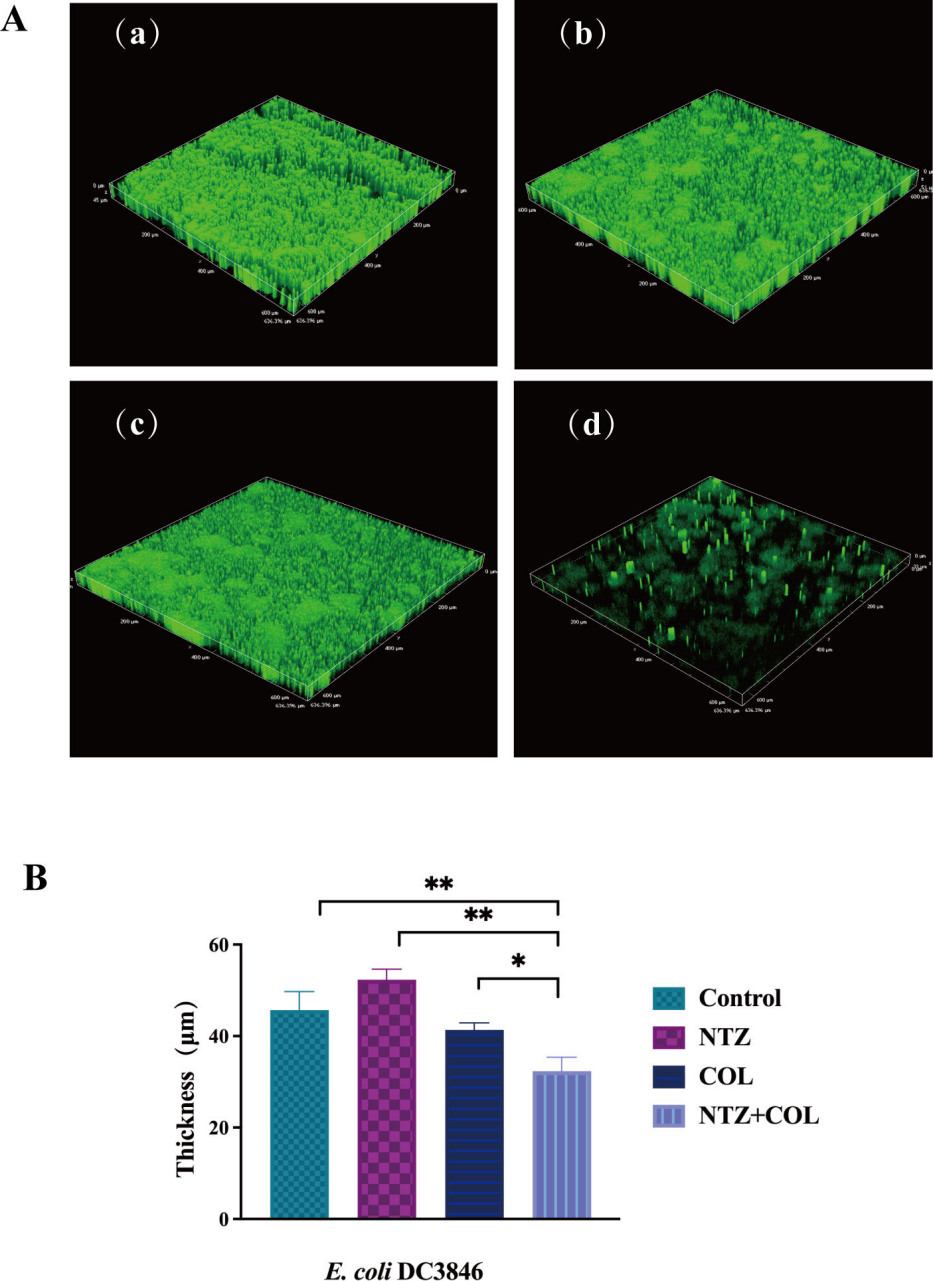
(A) CLSM images displaying the effect of different treatments on the number and structure of biofilm of COL-R *E. coli* DC3846. (a) LB broth control at 3D. (b) NTZ alone at 3D. (c) COL alone at 3D. (d) COL/NTZ combination at 3D. (**B**) CLSM images displaying the average thickness of COL-R *E. coli* DC3846 biofilm formation by different treatments. Data were analyzed by student’s *t*-test (*P < 0.05, **P < 0.01).

**FIG 6:**
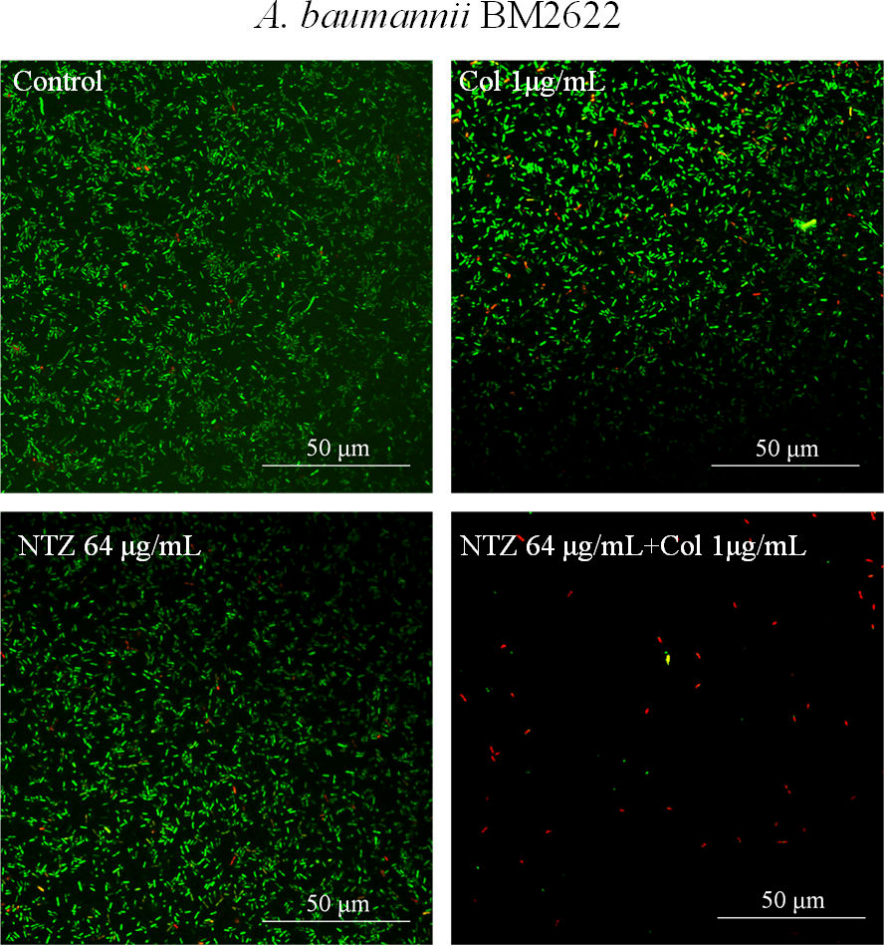
CLSM images of COL-R *A. baumannii* BM2622 biofilms in different treatment groups after live/dead staining. Live cells are stained green, and dead cells are stained red.

Microscopic examination using CLSM provided additional insights. The images revealed that the untreated *E. coli* cells exhibited a thick biofilm (Fig. 5a). The groups treated with 32 µg/mL NTZ or 1 µg/mL COL also formed abundant biofilms (Fig. 5b and c), whereas the combination of NTZ and COL significantly reduced biofilm biomass (Fig. 5d). In addition, quantification of biofilm thickness on CLSM imaging showed a reduction in the thickness and fluorescence intensity of biofilm formation after the combined administration of NTZ and COL compared to the control group (Fig. 5B). In addition, live/dead cell staining was used to observe the effect of the combination of NTZ and COL on cell viability. The results showed that there were a large number of live bacteria on the surface of the single drug treatment group (green light). In contrast, the number of viable bacteria on the surface was significantly reduced (red light) in the NTZ and COL combined treatment groups (Fig. 6).

### Synergistic therapeutic effects of COL and NTZ *in vivo*

**FIG 7:**
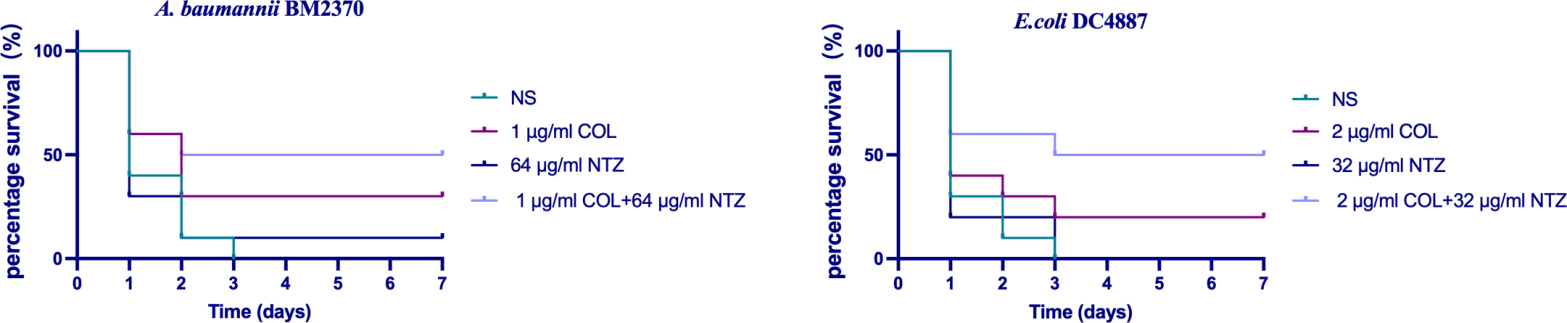
Survival rates of *G. mellonella* infected with COL-R *A. baumannii* BM2370 and COL-R *E. coli* DC4887 after seven days of monotherapy or combination treatments. NS: normal saline.

To determine the *in vivo* therapeutic effect of the COL/NTZ combination against COL-R bacteria, *G. mellonella* survival assay was conducted (Fig. 7). Without drug treatment, nearly all *G. mellonella* died after three days, and the survival rates of *G. mellonella* subjected to NTZ monotherapy were substantially lower than those subjected to combination therapy (*P* < 0.05). In the combination therapy group, a seven-day survival rate of 50% was observed among the tested stains. The combination of COL and NTZ was more effective than either drug used alone. The results suggest that the combination of the two drugs exerts good synergistic therapeutic effects on COL-R bacterial infections *in vivo*.

### *In vitro* hemolytic activity assay

**FIG 8:**
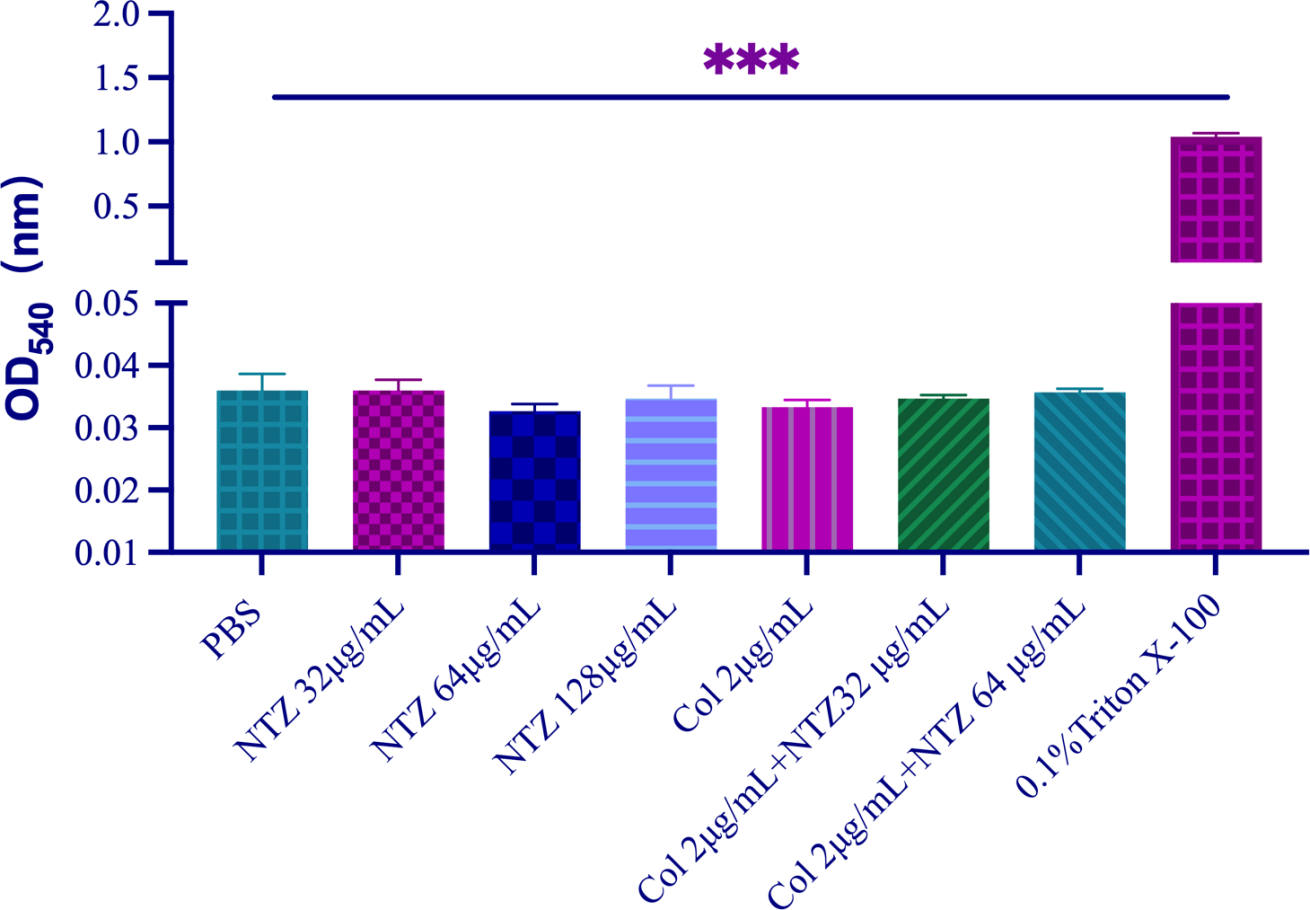
Hemolysis effect at different concentrations of NTZ and/or COL. PBS served as the negative control, while 0.1% Triton X-100 served as a positive control.

To verify the safety of NTZ in combination with COL, we investigated the toxic effects of NTZ alone and NTZ in combination with COL on red blood cells (RBCs). Phosphate-buffered saline (PBS)-treated erythrocytes were used as the negative control group, and 0.1% Triton X-100-treated erythrocytes were used as the positive control group. Fig. 8 shows that compared with the negative control PBS, NTZ monotherapy had no additional toxic effects on erythrocytes when used at the highest concentration of 128 µg/mL as well as at the highest combined concentration of 2 µg/mL COL + 64 µg/mL NTZ in this study. This further proves that the combination therapy is safe *in vivo*.

## DISCUSSION

COL-R Gram-negative bacteria are rapidly spreading worldwide, posing a substantial threat to public health and safety (11). Therefore, there is an urgent need for new therapeutic strategies to combat these superbugs. NTZ is an orally active drug with extensive post-marketing experience that is currently widely used in a variety of infectious diseases (12). NTZ can be utilized in combination with linezolid against infections caused by linezolid-resistant *S. aureus*, as it exhibits strong synergism *in vitro* and *in vivo* (13). In addition, NTZ exhibits broad-spectrum activity against anaerobes, parasites, and ulcer-causing *Helicobacter pylori* (7). The antibacterial effect of NTZ plus COL against Gram-negative bacteria has not been reported in the literature. Here, we used NTZ combined with COL *in vivo* and *in vitro* to develop new treatments against COL-R pathogens.

In antimicrobial susceptibility testing, NTZ monotherapy exhibits a high MICs (≥256 µg/mL), indicating ineffectiveness against COL-R Gram-negative strains, consistent with previous findings (13). The FICI in the presence of both NTZ and COL is in the range of 0.25–0.5. The MIC of NTZ significantly decreased by 4- to 8-fold and that of COL decreased by 4- to 64-fold (Table 2), indicating the significant effect of NTZ plus COL. We also explored the combined effect of NTZ and colistin against colistin-susceptible strains. The results indicated that NTZ combined with COL had no combined effect against colistin-susceptible strains. Moreover, it has been reported that colistin combined with antibiotics had no combined effect on COL-sensitive strains, but had a combined effect on colistin-resistant strains (14), which is consistent with our results. We speculated that this observation may be related to the complex COL resistance mechanism as well as the fact that the MIC value of colistin sensitivity cannot be reduced indefinitely. The neurotoxicity and nephrotoxicity of COL, as well as the emergence of COL-R strains, limit its clinical application (15). The combination of the two drugs can restore the sensitivity of COL and reduce its dosage.

This effect was further confirmed through time-killing assays under dynamic conditions mimicking clinical conditions. NTZ combined with COL showed significant bactericidal activity against COL-R *A. baumannii* and COL-R *E. coli* within 12 h and had a good synergistic antibacterial effect (Fig. 3A and B). The antimicrobial activity remained effective for 24 h against COL-R *A. baumannii* and COL-R *E. coli*, although the inhibitory effect was weakened after 24 h compared to 12 h. Therefore, it is necessary to further investigate the causes of this weakened synergistic effect of the combination after 12 h. This is thought to be attributable to the persistence of bacteria, consistent with previous results (16). Therefore, to completely kill the bacteria, a higher drug concentration or drug re-administration after 12 h is necessary.

The biofilm is a structured bacterial community that poses a significant challenge to the effectiveness of conventional antibiotics and is recognized as a breeding ground for antibiotic resistance. The National Institutes of Health estimates that more than 16 million people die annually because of bacterial infections worldwide (17). In addition, about 80% of them are associated with bacterial biofilm formation-mediated resistance (18). The results showed that COL combined with NTZ could effectively inhibit the biofilm formation of most experimental strains of COL-R *A. baumannii* and COL-R *E. coli*. In addition, based on the data from live/dead cell staining experiments, we found that the COL/NTZ combination could produce a more obvious effect on bacterial viability when compared to NTZ or COL alone. According to reports, NTZ inhibits the biofilm production of enteroaggregative *E. coli* strains by blocking the assembly of AafA fimbriae (19). Biofilm inhibition of the NTZ may be due to the inhibition of the motility of *E. coli*. Furthermore, the COL/NTZ combination disrupted the DC3846 biofilm and reduced cell alignment density under the SEM.

In recent reports, NTZ was shown to significantly eliminate existing *S. aureus* biofilms without the development of resistance to it by *S. aureus* (13). Removing preformed biofilms is more difficult than inhibiting them. Antibiotics are rarely administered prophylactically, but they target groups that are already resistant during infection. In this study, we conducted a biofilm eradication assay against COL-R *A. baumannii* and COL-R *E. coli*. The synergy between COL and NTZ in eradicating established strain biofilms highlights the promise of this antibacterial adjuvant regimen.

In addition, the efficacy of the combination was evaluated *in vivo* using the *G. mellonella* infection model. NTZ combined with COL enhanced the survival rates of *G. mellonella in vivo*. Furthermore, RBC cytotoxicity testing confirmed that the drug combination was not cytotoxic at combination concentrations. The *in vivo* application of this combination suggests a considerable level of safety. Pharmacologically, NTZ has an excellent safety and tolerability profile, with an LD_50_＞1,300 mg/kg in mice and demonstrating moderate absorption (6). Our work may help provide research directions for the reuse of existing drugs as adjuvants to expand the therapeutic range of these drugs.

### Conclusion

This study demonstrated for the first time that NTZ combined with COL exhibits a significant synergistic effect against COL-R *A. baumannii* and COL-R *E. coli* while reducing biofilm formation. In addition, this combination can reduce the dose of COL and restore its potential as a first-line antibacterial drug, providing a new option for clinical anti-infective treatment.

## MATERIALS AND METHODS

### Bacterial strains, medium, antibiotics, and solvents

A total of eight COL-R *A. baumannii* and eight COL-R *E. coli* non-duplicate isolates were isolated from the First Affiliated Hospital of Wenzhou Medical University during 2012–2019. All isolates were cultured on a Columbia blood agar plate (BAP). Strains were identified using matrix-assisted laser desorption/ionization time-of-flight mass spectrometry (MALDI-TOF-MS, BioMerieux, France) and stored at −80℃ in Luria Bertani (LB) broth medium supplemented with 30% glycerin (Thermo Fisher Scientific, Shanghai, China.).

### Antimicrobial susceptibility tests

*E. coli* ATCC 25922 and *P. aeruginosa* ATCC 27853 were used as quality control strains purchased from the National Center for Clinical Laboratories. Antibiotic solutions, including aztreonam, ceftazidime, cefepime, imipenem, ciprofloxacin, levofloxacin, gentamicin, tobramycin, and COL (Kangtai Biological Technology, Zhejiang, China), were prepared following the CLSI 2022 guideline. NTZ (MedChem Express, Co., Ltd. NJ, USA) was dissolved in 2.5% (vol/vol) dimethyl sulfoxide. The MICs of COL-R isolates to nine antibiotics and NTZ were measured using the broth microdilution method. Briefly, 0.5 MacFarland suspension was prepared from a single colony on BAP using sterile normal saline and further diluted to 10^6^ CFU/mL with cation-adjusted Mueller–Hinton broth (CAMHB). Wells were filled with continuous concentrations of antibiotics ranging from 0.06 to 128 µg/mL and NTZ 4–256 µg/mL, and 100 µL (10^5^ CFU/mL) of bacterial suspension were introduced into each well. The MIC value was defined as the lowest concentration that completely inhibited bacterial growth, as determined by visual inspection after incubating for 16–20 h at 37℃. The breakpoints of antibiotics were referred to CLSI 2022 (20). Each MIC test against all isolates was performed in duplicate.

### Checkerboard assays

Eight COL-R *A. baumannii* and eight COL-R *E. coli* isolates were employed for the checkerboard experiments, which examined the synergistic effects of twofold successive drug dilutions placed in a 12 × 8 matrix (21). COL and NTZ were diluted in CAMHB to various MIC values along the horizontal and longitudinal blanks of plates, and 10^5^ CFU/mL of bacteria were introduced. Results were observed after 16–20 h of incubation at 37°C. A microplate reader was used to determine the optical density of the bacterial culture at 600 nm. All experiments were performed in triplicate.

The FICI was used to evaluate the synergistic effect of NTZ plus COL. The formula for calculating FICI is as follows: FICI = (MIC of drug A in combination/MIC of drug A alone) + (MIC of drug B in combination/MIC of drug B alone), where FICI ≤0.5 indicates synergistic, FICI >0.5 and FICI ≤4 indicates irrelevantly, and FICI >4 is considered antagonistic (22).

### CV staining

The biofilm formation abilities of COL-R *A. baumannii* and COL-R *E. coli* were assessed via CV staining, as previously described and with minor modifications (23). A fresh single colony was cultured overnight. NTZ and COL were added to the 96-well plate at final concentrations of 32–64 µg/mL and 0.25–1 µg/mL, and 10^5^ CFU/mL of bacteria were introduced. Cells underwent static incubation for 24 h at 37℃ and were washed three times with PBS (pH = 7.2) to remove planktonic cells, followed by drying at 60℃. Staining with 0.1% CV for 30 min was performed, and the stained biofilm was washed with PBS to remove the remnants. The CV contained in biofilm was dissolved in 150 µL of 95% ethanol, and its absorbance at 595 nm was measured. The experiments were performed in triplicate.

### Preformed mature biofilm eradication assays

We based our methodology on previous reports, with minor modifications (24). Four COL-R *A. baumannii* and four COL-R *E. coli* isolates were randomly selected to evaluate the eradication effect of NTZ combined with COL on mature biofilms. First, individual colonies on blood plates were shaken overnight in LB broth medium. The bacterial suspension was adjusted to 0.5 McFarland standard using sterile saline and then diluted at a 1:100 ratio in LB broth. Second, 200 µL of bacterial suspension was added to each well of a 96-well plate and incubated at 37°C for 24 h to facilitate the formation of a mature biofilm. After incubation, the supernatant was discarded, and the plates were washed three times with 0.9% saline to remove unattached cells. Next, NTZ and COL were added to the single or combined groups at final concentrations of 128 µg/mL and 4 µg/mL, respectively, in 96-well plates and incubated for 24 h at 37°C. The staining procedure was the same as described above. The experiments were performed in triplicate.

### Time-kill assay

Bacterial cultures were prepared as described for combination experiments (25). Briefly, 1 × 10^6^ CFU/mL of bacteria were exposed to NTZ and COL alone or in combination. Bacteria without antibiotic treatment served as a control for growth. The suspensions were incubated at 37°C with moderate shaking for 24 h. CFU counting was performed at 0, 4, 8, 12, and 24 h. Bactericidal activity was defined as a reduction of ≥3 log_10_ CFU/mL at 24 h and synergistic activity was defined as a reduction of ≥2 log_10_ CFU/mL at 24 h in the presence of the two-drug combination compared to each drug administered individually.

### Scanning electron microscope

SEM was used to demonstrate the effects of the COL/NTZ combination on the biofilm of *E. coli* DC3846. *E. coli* DC3846 was cultured overnight and adjusted to 0.5 McFarland standard using sterile NS. The control, NTZ monotherapy, COL monotherapy, and combination groups were established. A sterile round glass slide was placed in each well, and 10^7^ CFU bacteria were added to LB broth (2 mL) containing 1 µg/mL of COL or 32 µg/mL of NTZ. The cultures were kept at 37°C for 18–24 h. Subsequently, the glass slides were removed, washed three times with distilled water, and fixed overnight with 2.5% (vol/vol) glutaraldehyde. After air drying, the samples were treated with gold sputtering and observed under an SEM (S-3000N, Japan) (26).

### Confocal laser scanning microscopy (CLSM)

In line with a previous protocol, we analyzed the *E. coli* biofilms using CLSM. In brief, *E. coli* DC3846 (10^6^ CFU/mL) was inoculated in LB broth containing 1 µg/mL of COL, 32 µg/mL of NTZ, and 1 µg/mL of COL + 32 µg/mL of NTZ to form static biofilms on coverslips for 24 h at 37°C in the six-well plates. Biofilms were washed twice with PBS (pH = 7.2) before removing planktonic cells and stained using SYTO 9 (L7012, Invitrogen, Thermo Fisher Scientific, Eugene, OR, USA) according to the procedure. Then, excess staining was eliminated by washing twice with PBS (pH = 7.2), and the biofilm was imaged using CLSM (LSM800, Zeiss, Jena, Germany).

In addition, COL-R *A. baumannii* BM2622 was randomly selected for live/dead staining experiments. *A. baumannii* BM2622 (10^6^ CFU/mL) was inoculated in LB broth containing 1 µg/mL COL, 64 µg/mL NTZ, and 1 µg/mL COL plus 64 µg/mL NTZ to form static biofilms on coverslips for 24 h at 37°C in six-well plates. Biofilms were stained using the SYTO-9/PI live/dead bacteria double staining kit (Fushenbio, Shanghai, China), which stained live bacterial DNA green and dead cells red due to PI staining. The excess staining solution was then removed by washing twice with PBS, and the specimens were observed using CLSM (LSM800, Zeiss, Jena, Germany).

### *Galleria mellonella* infection model

The *in vivo* synergy of COL and NTZ was evaluated using modified survival assays involving *G. mellonella* infected with *A. baumannii* and *E. coli*, as previously described (27). The overnight cultures of *A. baumannii* BM2370 and *E. coli* DC4887 were adjusted to 1.5 × 10^5^ CFU/mL using sterile normal saline. The bacterial solution (10 µL) was injected into the larva’s left hind limb using a microsyringe and treated with the test drug alone or in a combination of 7× MICs after 1 h of infection. Larvae treated with normal saline served as positive controls. Larvae were aerobically incubated at 37°C for seven days, and the survival rates of *G. mellonella* were recorded every 24 h. All experiments were in triplicate. Larvae with darkened bodies and no reaction to repeated physical stimuli were considered dead.

### Hemolysis test

The hemolytic effect of NTZ was assessed using RBCs from healthy volunteers, as described previously (28). Blood samples were collected in EDTA-2Na tubes and centrifuged at 3,000 rpm for 5 min, after which serum was removed and RBCs were resuspended thrice in PBS. Then, 6% (vol/vol) of the RBC suspension was diluted with PBS. The RBC suspension was mixed with isopycnic NTZ at a concentration of 0–128 μg/mL. After 1 h of incubation at 37℃, suspensions were centrifuged at 3,000 rpm for 5 min, and the OD_540nm_ of the supernatant was determined using a microplate reader. RBCs treated with PBS and 0.1% Triton X-100 served as negative and positive controls, respectively.

### Statistical analysis

Comparison between the two groups was performed using no-paired Student *t*-tests or non-parametric Mann–Whitney test, while multiple comparisons were made using one-way analysis of variance with *post hoc* analysis using Tukey’s test in GraphPad Prism (GraphPad Prism 8.0.1, GraphPad, San Diego, CA, USA). The results from all experiments were presented as the mean ± SD of three replicates.

## Data Availability

The data sets generated for this study are available on request to the corresponding author.
